# Poor Distal Left Atrial Appendage Opacification on Early-Phase Cardiac CTA and Association With Arterial Thromboembolism

**DOI:** 10.31083/RCM47945

**Published:** 2026-06-25

**Authors:** Tao He, Cong Lu, Tiantian Luo, Jie Zeng

**Affiliations:** ^1^Department of Cardiology and Center of Structure Heart Disease, Sichuan Provincial People’s Hospital, University of Electronic Science and Technology of China, 610072 Chengdu, Sichuan, China; ^2^School of Medical and Life Sciences, Chengdu University of Traditional Chinese Medicine, 611137 Chengdu, Sichuan, China

**Keywords:** atrial fibrillation, atrial appendage, computed tomography angiography, thromboembolism, stroke

## Abstract

**Background:**

Poor contrast opacification (PCO) of the distal left atrial appendage (LAA) on early-phase cardiac computed tomography angiography (CTA) may reflect impaired LAA hemodynamics and blood stasis, potentially predisposing to thrombus formation and embolic events. Thus, this study aimed to evaluate the association between early-phase LAA-PCO and arterial thromboembolic events in patients with non-valvular atrial fibrillation (AF).

**Methods:**

In this single-center retrospective cohort study, 389 consecutive adult patients with non-valvular AF who underwent cardiac CTA were analyzed between January 2020 and December 2023. Qualitative LAA-PCO on early-phase CTA and quantitative Hounsfield unit (HU) measurements at the LAA ostium, mid-body and distal LAA, left atrium (LA), and descending aorta (DA) were recorded. The O/D ratio (LAA-ostium HU/distal LAA HU) and a corrected O/D ratio normalized by LA/DA attenuation were calculated. Associations with ischemic stroke and other arterial embolic events were assessed using multivariate logistic regression, and model discrimination was assessed using the C-index.

**Results:**

Of the 389 patients, 104 experienced arterial embolic events, including 102 ischemic strokes. LAA-PCO was present in 68 patients (17.48%) and was more common in the embolic group (27.88% vs. 13.68%, *p* = 0.001). The embolic group had significantly lower distal LAA HU and higher O/D and corrected O/D ratios. In the multivariate analysis, the CHA_2_DS_2_-VASc score (odds ratio (OR) = 2.442; *p* = 0.001), LAA-PCO (OR = 2.120; *p* = 0.014), O/D ratio (OR = 1.250; *p* = 0.004) and corrected O/D ratio (OR = 1.325; *p* = 0.002) were independently associated with arterial embolism. A model combining the CHA_2_DS_2_-VASc score and the corrected O/D ratio showed a relatively high C-index of 0.87.

**Conclusions:**

Poor distal LAA opacification on early-phase CTA is associated with arterial thromboembolism in patients with non-valvular AF and may provide complementary information to the CHA_2_DS_2_-VASc score for thromboembolic risk assessment. Prospective validation and standardization of computed tomography (CT) protocols are needed before clinical implementation.

## 1. Introduction

Atrial fibrillation (AF) is one of the most prevalent cardiac arrhythmias encountered in clinical practice, and thromboembolic events are its most serious complication. Patients with non-valvular atrial fibrillation (NVAF) face an approximately fivefold higher risk of ischemic stroke compared with the general population [1]. Accurate stratification of thromboembolic risk is therefore essential for effective prevention and management; nevertheless, reliable risk prediction remains a major clinical challenge. The widely used CHA_2_DS_2_-VASc score, which is based solely on clinical variables, provides only modest discriminatory performance for stroke prediction (C-statistic ≈ 0.6) [2,3].

The left atrial appendage (LAA) is the dominant source of thrombus formation in NVAF, accounting for the vast majority of cardioembolic events [4,5]. Morphologic, structural, and hemodynamic characteristics of the LAA have been shown to be associated with the risk of stroke independent of conventional clinical scores [6,7,8]. Cardiac computed tomography angiography (CTA) has been increasingly employed to evaluate LAA anatomy and to detect filling defects, offering higher spatial resolution than transesophageal echocardiography (TEE) for certain assessments [9,10].

Recent studies have reported that poor contrast opacification (PCO) of the distal LAA on early-phase CTA is associated with an elevated risk of ischemic stroke, likely reflecting impaired LAA contractile function and intra-appendage blood stasis—conditions that predispose to thrombus formation [11,12,13,14]. However, prior studies have generally been limited by small sample sizes and heterogeneous imaging protocols, and the association between early-phase LAA-PCO and clinical thromboembolic outcomes has not been consistently reproduced across various study populations [11,13].

Accordingly, there is a need for larger sample studies that systematically evaluate early-phase LAA filling on CTA and clarify its clinical relevance in relation to arterial thromboembolism in patients with AF. The present retrospective analysis addresses this gap by examining a large cohort of AF patients who underwent cardiac CTA, quantifying early-phase LAA opacification, and assessing the relationship between LAA-PCO and subsequent thromboembolic events to determine whether CTA-derived LAA metrics provide complementary discriminative information beyond established clinical risk scores.

## 2. Materials and Methods

### 2.1 Study Population and Grouping

This single-center, retrospective cross-sectional study enrolled consecutive adult patients (≥18 years) with non-valvular atrial fibrillation (paroxysmal, persistent, or long-standing persistent) who were admitted and scheduled for LAA occlusion or radiofrequency ablation between January 2020 and December 2023. Exclusion criteria were: (1) valvular atrial fibrillation (AF associated with moderate or severe mitral stenosis); (2) congenital heart disease with major structural defects (e.g., atrial septal defect); (3) severe infectious diseases; (4) prior cardiac surgery (e.g., valve replacement); (5) severe hepatic or renal dysfunction; (6) allergy to iodinated contrast media; and (7) missing or non-diagnostic cardiac CTA images.

A total of 415 patients were identified from the hospital medical records. After excluding 4 patients with incomplete clinical data and 22 patients whose cardiac CTA images were unsuitable for analysis, 389 patients were included in the final cohort. Patients were divided into an embolic group (n = 104) and a non-embolic group (n = 285) according to the presence or absence of arterial embolic events. Embolic events included ischemic stroke and other systemic arterial embolism (e.g., mesenteric or peripheral arterial embolism). Ischemic stroke was diagnosed by a neurologist based on characteristic clinical symptoms and corroborating cranial imaging; the presence of an infarct on cranial CT or magnetic resonance imaging (MRI) defined ischemic stroke. Embolic events at other sites required compatible imaging and clinical findings for diagnosis.

### 2.2 Clinical Data Collection

All clinical and imaging data used in this study were collected prior to the intervention. Demographic and clinical variables included sex, age, AF type, AF duration, and comorbidities. Laboratory data included high-sensitivity troponin I (hs-TnI), B-type natriuretic peptide (BNP), platelet count (PLT), hemoglobin (HGB), white blood cell count (WBC), total bilirubin (TBIL), serum creatinine (Cr), and estimated glomerular filtration rate (eGFR). Clinical risk scores (CHA_2_DS_2_-VASc and HAS-BLED) and symptom severity (European Heart Rhythm Association score, EHRA score) were recorded. Transthoracic echocardiography was used to determine chamber dimensions and left ventricular ejection fraction (LVEF). TEE reports were reviewed for the presence of LAA thrombus, spontaneous echo contrast (SEC), and the velocity of LAA emptying.

### 2.3 Cardiac CTA Acquisition

Cardiac CTA examinations were performed using either a Siemens Dual-Source Force scanner (Siemens Healthcare GmbH, Forchheim, Germany) or a GE Revolution 256-slice CT scanner (GE Medical Systems, LLC, Waukesha, WI, USA). Patients were scanned in the supine position using retrospective ECG gating and instructed to perform a single breath-hold at end-expiration. Bolus tracking triggering was used in this study, with image acquisition initiated when the attenuation within a predefined region of interest (ROI) in the ascending aorta exceeded 100 Hounsfield units. Iodinated contrast (350 mg I/mL) was administered with a power injector at 4–5 mL/s at a dose of 0.8 mL/kg, followed by a 30 mL saline flush at the same injection rate. Typical scan parameters were 80–120 kV tube voltage, 280–350 mAs per rotation, collimation 128 × 0.625 mm, pitch 0.18, rotation time ~330 ms per rotation, image matrix 512 × 512, and field of view ≈ 250 mm.

### 2.4 Image Post-Processing and Analysis

**Fig. 1.**
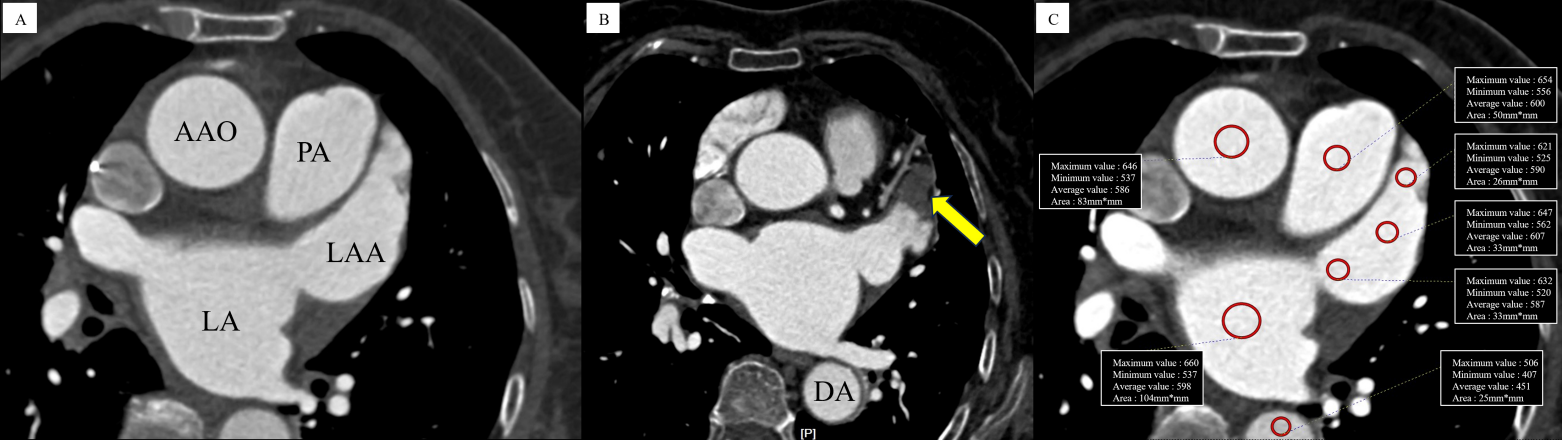
**Contrast filling pattern and measurement method of LAA**. (A) demonstrates uniform contrast opacification of the left atrial appendage. (B) shows inadequate contrast opacification in the distal left atrial appendage. The yellow arrow denotes poor contrast opacification. (C) shows the measurement of Hounsfield unit (HU) values at different regions. AAO, ascending aorta; PA, pulmonary artery; LAA, left atrial appendage; LA, left atrium; DA, descending aorta.

All image analyses were performed on the Neusoft PACS/RIS imaging system (Neusoft, Shenyang, China; version 5.5). Early-phase diastolic axial source images were used for assessment. PCO of the distal LAA (LAA-PCO) was defined qualitatively as a low-attenuation region visible in the distal LAA on early-phase images, where contrast opacification of the distal LAA is markedly lower than that of the left atrium (LA) and the LAA ostium—representing incomplete mixing of contrast and blood in the early phase (Fig. 1A,B).

Quantitative measurements of Hounsfield units (HU) were obtained at the following sites: LAA ostium (LAA-O), LAA body/mid portion (LAA-M), LAA distal portion (LAA-D), LA, and descending aorta (DA). The O/D ratio was calculated as LAA-O HU divided by LAA-D HU (O/D ratio = LAA-O/LAA-D). Because early-phase LAA opacification may be influenced by contrast arrival time and systemic factors, a corrected O/D ratio was also computed to normalize for systemic contrast enhancement: Corrected O/D ratio = (LAA-O/LAA-D)/(LA HU/DA HU) (Fig. 1C).

Two interventional cardiologists jointly collected imaging measurements for subsequent analysis. To assess reproducibility, 20 patients were randomly selected for inter-observer variability analysis. If there was disagreement between the two readers in qualitative interpretation, a third, more senior clinician adjudicated the findings.

### 2.5 Statistical Analysis

Statistical analyses were conducted using IBM SPSS Statistics version 27 (IBM Corp., Armonk, NY, USA). Continuous variables are presented as mean ± standard deviation, and categorical variables as percentages. Between-group comparisons for normally distributed continuous variables with equal variances were performed using Student’s *t*-test, whereas non-normally distributed continuous variables were compared using rank-sum tests. Categorical variables were compared using the chi-square test. Univariate and multivariate binary logistic regression analyses were used to identify factors associated with thromboembolic events. Missing data were imputed using the variable mean or median. Variables with >20% missing data were excluded from the analysis.

Reproducibility was evaluated via the Bland–Altman analysis. Intraclass correlation coefficient (ICC) together with the coefficient of variation (CV). ICC values >0.90 were considered excellent, 0.75–0.90 as good, 0.50–0.75 as moderate, and <0.50 as poor. Spearman correlation was used for two-variable correlation analyses. The C-index was used to compare the discriminatory performance of CHA_2_DS_2_-VASc alone versus models combining CHA_2_DS_2_-VASc with LAA HU metrics for stroke discrimination. A two-sided *p*-value < 0.05 was considered statistically significant.

## 3. Results

### 3.1 Baseline Characteristics

**Fig. 2.**
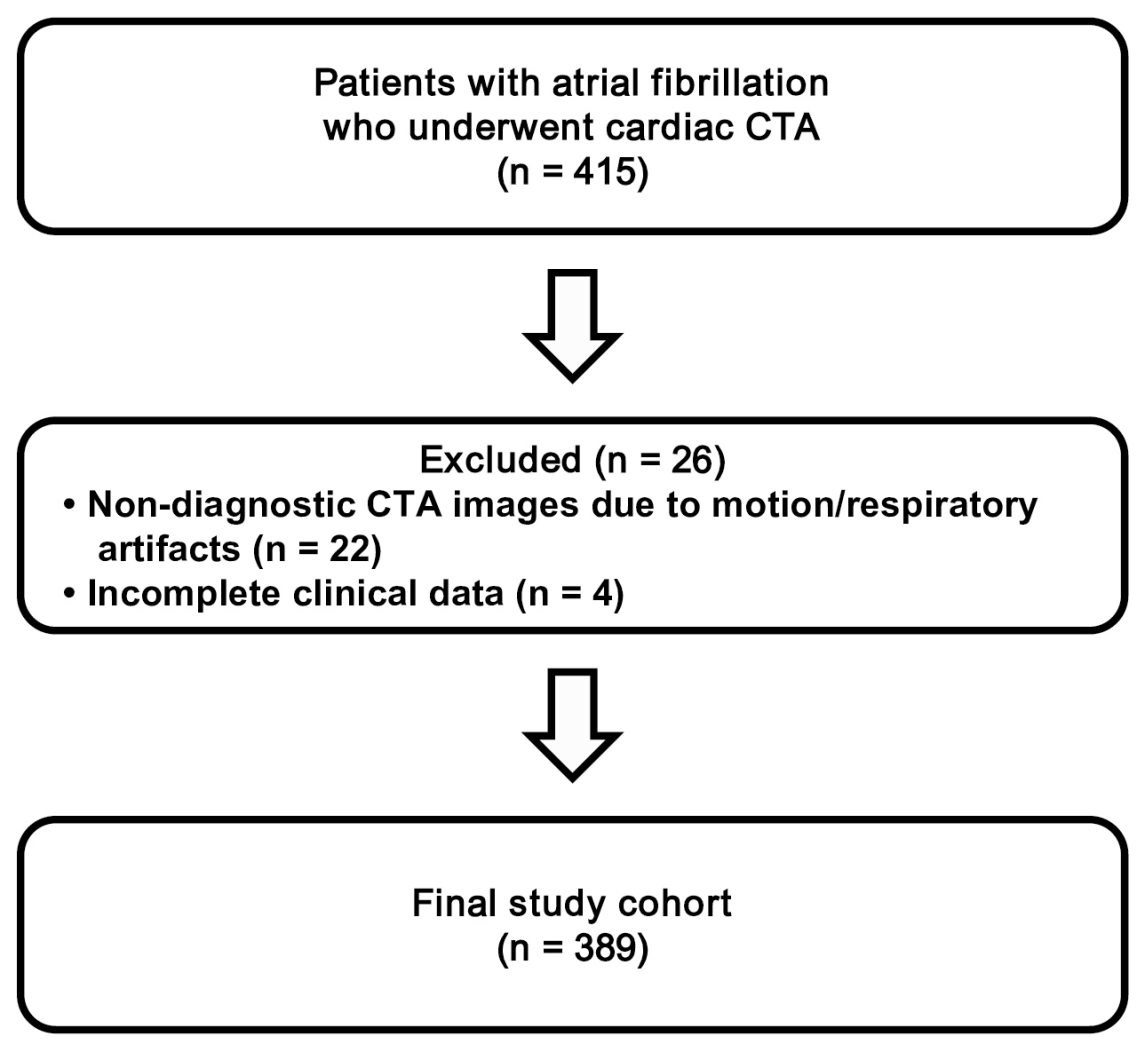
**Flowchart of patient screening and selection**. CTA, computed tomography angiography.

A total of 389 patients with non-valvular atrial fibrillation were included in the study, of whom 104 were in the embolic group and 285 in the non-embolic group. Fig. 2 shows the flowchart for patient screening and inclusion. In the embolic group, 99 patients had ischemic stroke, 3 patients had both ischemic stroke and limb arterial embolism, and 2 patients had isolated limb arterial embolism. Patients in the embolic group were older (69.79 ± 8.27 vs. 63.15 ± 11.54 years, *p* = 0.001). The embolic group had a relatively lower proportion of paroxysmal AF (33.7% vs. 41.4%, *p* = 0.197), a significantly higher prevalence of hypertension (67.3% vs. 49.8%, *p* = 0.003) and diabetes (27.9% vs. 18.6%, *p* = 0.050). The embolic group also exhibited larger left atrial diameter (42.80 ± 5.95 vs. 40.71 ± 6.61 mm, *p* = 0.005) and right atrial diameter (52.28 ± 7.15 vs. 50.01 ± 8.43 mm, *p* = 0.015), and higher platelet count (180.86 ± 61.68 vs. 167.14 ± 61.91 ×10^9^/L, *p* = 0.054). The embolic group had higher CHA_2_DS_2_-VASc scores (4.67 ± 1.48 vs. 2.31 ± 1.58, *p* = 0.001) and higher HAS-BLED scores (2.06 ± 0.80 vs. 0.84 ± 0.78, *p* = 0.001), but lower EHRA symptom scores (2.10 ± 0.84 vs. 2.42 ± 0.65, *p* = 0.001). Preprocedural LAA emptying velocities were available in 58 patients; velocities were lower in the embolic group, but did not reach statistical significance (0.37 ± 0.21 vs. 0.50 ± 0.26 m/s, *p* = 0.059). Detailed baseline characteristics are described in Table 1.

**Table 1.** **Comparison of baseline characteristics between groups**.

Variable		Total (n = 389)	Embolic group (n = 104)	Non-embolic group (n = 285)	*p* value
Age (years)		64.93 ± 11.15	69.79 ± 8.27	63.15 ± 11.54	0.001
Male, n (%)		213 (54.8)	57 (54.8)	156 (54.7)	0.990
AF type	Paroxysmal, n (%)	153 (39.3)	35 (33.7)	118 (41.4)	0.197
	Persistent, n (%)	140 (36.0)	41 (39.4)	99 (34.7)	0.405
	Long-standing persistent, n (%)	96 (24.7)	28 (26.9)	68 (23.9)	0.595
Coronary artery disease, n (%)		65 (16.7)	19 (18.4)	46 (16.1)	0.644
Diabetes, n (%)		82 (21.1)	29 (27.9)	53 (18.6)	0.050
Hypertension, n (%)		212 (54.5)	70 (67.3)	142 (49.8)	0.003
Chronic HF, n (%)		55 (14.1)	9 (8.7)	46 (16.1)	0.061
Anticoagulation, n (%)		80 (20.6)	27 (26.0)	53 (18.6)	0.112
EHRA score		2.33 ± 0.72	2.10 ± 0.84	2.42 ± 0.65	0.001
CHA_2_DS_2_-VASc score		2.94 ± 1.88	4.67 ± 1.48	2.31 ± 1.58	0.001
HAS-BLED score		1.16 ± 0.95	2.06 ± 0.80	0.84 ± 0.78	0.001
LV (mm)		46.16 ± 6.26	45.79 ± 4.51	46.30 ± 6.79	0.475
LA (mm)		41.26 ± 6.50	42.80 ± 5.95	40.71 ± 6.61	0.005
RV (mm)		21.55 ± 4.11	21.41 ± 3.44	21.59 ± 4.32	0.703
RA (mm)		50.62 ± 8.15	52.28 ± 7.15	50.01 ± 8.43	0.015
LVEF		0.64 ± 0.09	0.64 ± 0.08	0.65 ± 0.10	0.837
LAA emptying velocity (m/s)		0.48 ± 0.19 (n = 58)	0.37 ± 0.21 (n = 9)	0.50 ± 0.26 (n = 49)	0.059
hs-TnI (ng/L)^*^		4.5 (2.3–11.9)	4.5 (2.8–9.3)	4.5 (2.2–12.1)	0.498
BNP (pg/mL)^*^		120.8 (62.2–241.0)	120.8 (75.1–206.6)	120.8 (57.6–259.4)	0.768
PLT (×10^9^/L)		170.89 ± 62.07	180.86 ± 61.68	167.14 ± 61.91	0.054
HGB (g/L)		135.28 ± 19.38	132.92 ± 19.74	136.13 ± 19.21	0.148
WBC (×10^9^/L)		6.66 ± 2.02	6.66 ± 1.72	6.67 ± 2.12	0.958
TBIL (μmol/L)^*^		16.2 (12.1–20.6)	14.8 (11.0–19.5)	16.7 (12.5–20.9)	0.015
Cr (μmol/L)		73.85 ± 30.01	75.24 ± 31.04	73.34 ± 29.66	0.583
eGFR (mL/min/1.73 m^2^)		86.71 ± 19.21	83.69 ± 17.06	87.81 ± 19.85	0.061

AF, atrial fibrillation; HF, heart failure; EHRA score, European Heart Rhythm Association score; LV, left ventricle; LA, left atrium; RV, right ventricle; RA, right atrium; LVEF, left ventricular ejection fraction; LAA, left atrial appendage; hs-TnI, high-sensitivity troponin I; BNP, B-type natriuretic peptide; PLT, platelet; HGB, hemoglobin; WBC, white blood cell; TBIL, total bilirubin; Cr, creatinine; eGFR, estimated glomerular filtration rate. ^*^: Non-normally distributed variables are expressed as median (interquartile range).

### 3.2 Intra- and Inter-Observer Agreement

Twenty patients were randomly selected for reproducibility analysis of cardiac CTA measurements. For each measured site, both intra-observer and inter-observer ICC showed high agreement (>0.9), indicating excellent repeatability and reliability of the imaging measurements (Table 2).

**Table 2.** **Intra- and inter-observer ICC for HU measurements**.

Variable	Intra-observer ICC	Inter-observer ICC
LAA-O HU	0.967 (0.920–0.988)	0.982 (0.957–0.993)
LAA-M HU	0.978 (0.950–0.992)	0.961 (0.905–0.984)
LAA-D HU	0.917 (0.806–0.966)	0.984 (0.961–0.994)
LA HU	0.979 (0.948–0.991)	0.975 (0.940–0.990)
DA HU	0.977 (0.943–0.991)	0.952 (0.885–0.981)

ICC, intraclass correlation coefficient; LAA-O, ostium of left atrial appendage; LAA-M, mid portion of left atrial appendage; LAA-D, distal portion of left atrial appendage; LA, left atrium; DA, descending aorta; HU, Hounsfield unit.

### 3.3 CTA Image Analysis

On qualitative CTA assessment, LAA-PCO was observed in 68 patients (17.48% of the cohort). The prevalence of LAA-PCO was significantly higher in the embolic group than in the non-embolic group (27.88% vs. 13.68%, *p* = 0.001). Quantitatively, the Embolic group had significantly lower distal LAA HU (LAA-D) than the non-embolic group. HU values at the LA, LAA-O, LAA-M, and DA were higher in the embolic group compared with the non-embolic group. The O/D ratio (1.18 [1.01–2.89] vs. 1.03 [0.96–1.31], *p* = 0.001) and the corrected O/D ratio (1.13 [0.98–2.50] vs. 1.03 [0.93–1.32], *p* = 0.001) were both significantly higher in the embolic group (Table 3).

**Table 3.** **Measurement and analysis of left atrial appendage parameters on CTA**.

Variable	Total (n = 389)	Embolic group (n = 104)	Non-embolic group (n = 285)	*p* value
LAA-PCO, n (%)	68 (17.48%)	29 (27.88%)	39 (13.68%)	0.001
LAA-O HU	426.16 ± 86.51	452.38 ± 91.08	416.84 ± 83.02	0.001
LAA-M HU	418.02 ± 83.44	435.60 ± 84.20	411.77 ± 82.42	0.013
LAA-D HU	338.87 ± 141.72	303.72 ± 163.21	351.37 ± 131.32	0.003
LA HU	420.45 ± 93.05	445.02 ± 94.61	411.71 ± 91.07	0.002
DA HU	397.46 ± 76.44	415.40 ± 78.27	391.08 ± 74.89	0.006
O/D ratio^*^	1.06 (0.97–1.60)	1.18 (1.01–2.89)	1.03 (0.96–1.31)	0.001
Corrected O/D ratio^*^	1.06 (0.94–1.50)	1.13 (0.98–2.50)	1.03 (0.93–1.32)	0.001

CTA, computed tomography angiography; LAA-PCO, PCO of the distal LAA; LAA-O, ostium of left atrial appendage; LAA-M, mid portion of left atrial appendage; LAA-D, distal portion of left atrial appendage; LA, left atrium; DA, descending aorta; HU, Hounsfield unit; O/D ratio, LAA-O HU/LAA-D HU; corrected O/D ratio, (LAA-O/LAA-D)/(LA HU/DA HU); PCO, poor contrast opacification. ^*^: Non-normally distributed variables are expressed as median (interquartile range).

### 3.4 Risk Factors for Arterial Embolism

On univariate analysis, age, diabetes, hypertension, EHRA score, CHA_2_DS_2_-VASc score, HAS-BLED score, LA, RA, TBIL, and LAA-D HU values were significantly associated with embolic events (**Supplementary Table 1**). Multivariate analyses demonstrated that CHA_2_DS_2_-VASc score (OR = 2.442; 95% CI = 2.0–2.982; *p* = 0.001), LAA-PCO (OR = 2.120; 95% CI = 1.163–3.865; *p* = 0.014), O/D ratio (OR = 1.250; 95% CI = 1.074–1.454; *p* = 0.004), and corrected O/D ratio (OR = 1.325; 95% CI = 1.107–1.586; *p* = 0.002) remained independently associated with stroke and systemic arterial embolism (Table 4).

**Table 4.** **Multivariate logistic regression analysis**.

Variable	OR	95% CI (lower)	95% CI (upper)	*p* value
CHA_2_DS_2_-VASc	2.442	2.0	2.982	0.001
LA (mm)	0.973	0.923	1.026	0.315
RA (mm)	1.032	0.990	1.076	0.134
TBIL	0.963	0.925	1.003	0.071
LAA-PCO	2.120	1.163	3.865	0.014
O/D ratio	1.250	1.074	1.454	0.004
Corrected O/D ratio	1.325	1.107	1.586	0.002

OR, odds ratio; CI, confidence intervals; LA, left atrium; RA, right atrium; TBIL, total bilirubin; LAA-PCO, PCO of the distal LAA; O/D ratio, LAA-O HU/LAA-D HU; corrected O/D ratio, (LAA-O/LAA-D)/(LA HU/DA HU); PCO, poor contrast opacification.

### 3.5 Correlation Between LAA Emptying Velocity and CTA Metrics

A total of 376 patients underwent TEE; LAA thrombus was identified in 3 patients, and SEC in 8 patients. Among these 11 patients, 7 (63.6%) had apparent LAA-PCO on CTA. Preprocedural LAA emptying velocity was measured by TEE in 58 patients. Analyses in this small number of patients revealed that LAA emptying velocity correlated positively with LAA distal HU (ρ = 0.418, *p* = 0.001) and negatively with CHA_2_DS_2_-VASc score (ρ = –0.313, *p* = 0.017) and Corrected O/D ratio (ρ = –0.4, *p* = 0.002) (**Supplementary Table 2**). LAA emptying velocity was lower in patients with a higher corrected O/D ratio (≥2) than in those with a lower corrected O/D ratio (1 ~ <2) (0.352 ± 0.156 vs. 0.480 ± 0.200 m/s, *p* = 0.001) (**Supplementary Table 3**).

### 3.6 Incremental Discriminative Value of CTA-Derived Metrics

**Fig. 3.**
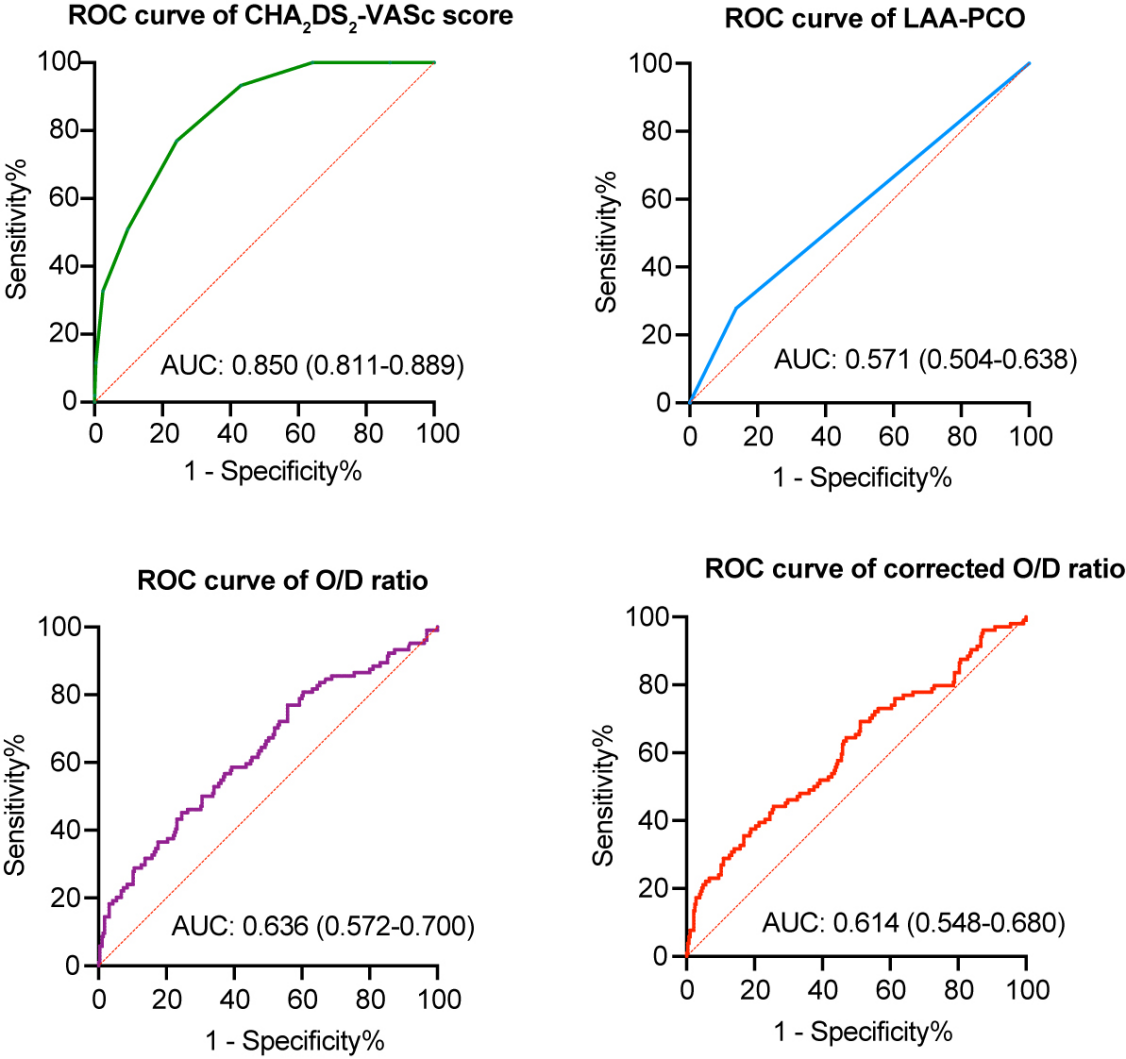
**Receiver operating characteristic curve of parameters**. This figure shows the ROC curve of the CHA_2_DS_2_-VASc score, LAA-PCO, O/D ratio, and corrected O/D ratio. Values in parentheses show the 95% confidence interval. ROC, receiver operating characteristic; AUC, area under ROC curve; LAA-PCO, PCO of the distal LAA; O/D ratio, LAA-O HU/LAA-D HU; corrected O/D ratio, (LAA-O/LAA-D)/(LA HU/DA HU).

Receiver operating characteristic (ROC) curve analysis showed that the CHA_2_DS_2_-VASc score demonstrated the highest area under the curve (AUC = 0.850). The O/D ratio (AUC = 0.636) and Corrected O/D ratio (AUC = 0.614) showed superior discrimination compared with qualitative LAA-PCO (AUC = 0.571) (Fig. 3). Using the Youden index, a corrected O/D ratio of 1.59 yielded a sensitivity of 0.375 and a specificity of 0.811, whereas an O/D ratio of 1.34 yielded a sensitivity of 0.45 and a specificity of 0.75. However, these thresholds should be considered exploratory and require prospective validation.

Incorporation of LAA-PCO and O/D measures improved discrimination for arterial embolism beyond the CHA_2_DS_2_-VASc alone. The model combining CHA_2_DS_2_-VASc score with the corrected O/D ratio achieved the highest C-index (C = 0.87), compared with CHA_2_DS_2_-VASc score alone (C = 0.85) (Table 5).

**Table 5.** **Model discrimination (C-index) for arterial thromboembolism**.

Model	C-index	95% CI (lower)	95% CI (upper)
CHA_2_DS_2_-VASc score	0.85	0.81	0.89
CHA_2_DS_2_-VASc + LAA-PCO	0.86	0.82	0.89
CHA_2_DS_2_-VASc + O/D ratio	0.86	0.83	0.90
CHA_2_DS_2_-VASc + corrected O/D ratio	0.87	0.83	0.90

LAA-PCO, PCO of the distal LAA; O/D ratio, LAA-O HU/LAA-D HU; corrected O/D ratio, (LAA-O/LAA-D)/(LA HU/DA HU).

## 4. Discussion

In this relatively large, single-center, retrospective cross-sectional analysis, we found that poor distal LAA opacification on early-phase cardiac CTA was independently associated with arterial embolic events in patients with non-valvular atrial fibrillation, even after adjustment for CHA_2_DS_2_-VASc score and other clinical covariates. Quantitatively, elevated O/D ratio (and corrected O/D ratio) reflecting a marked attenuation gradient between the LAA ostium and distal LAA, were significantly associated with embolic events, and these CTA metrics showed a negative correlation with LAA emptying velocity measured by TEE. These results indicate that early-phase LAA opacification abnormalities likely reflect impaired LAA hemodynamics and blood stasis, which may mechanistically contribute to thrombus formation and embolization.

Cardiac CTA is now widely used for peri-procedural evaluation of the LAA in patients with AF. Although delayed imaging provides excellent diagnostic performance for differentiating true thrombus from contrast stasis [14,15,16], it is not routinely acquired in clinical practice, and the prevalence of persistent, true LAA thrombus is relatively low. Consequently, its clinical value in predicting thromboembolic risk is limited. Previous studies have reported a relatively high prevalence of early-phase poor LAA opacification in AF patients, ranging from approximately 27.7% to 49% [11,12,17]. Inoue and Suematsu [11] first described an association between poor LAA enhancement on cardiac CT and stroke risk in patients with persistent AF, and subsequent single-center series have reported similar associations between early-phase LAA filling defects and prior ischemic events [12,13,17]. However, most earlier studies were limited by small sample sizes and relied on qualitative assessment of filling defects. The absence of standardized criteria for defining “poor opacification” introduced potential subjectivity and interobserver variability. Our study extends these observations by using a larger sample, providing objective, reproducible quantitative HU measurements (O/D ratio and corrected O/D ratio) with high inter- and intra-observer agreement, and demonstrating that CTA-derived LAA metrics add complementary discriminative information beyond CHA_2_DS_2_-VASc alone.

The pathophysiologic link between poor early opacification and thromboembolism is biologically plausible. Reduced LAA contractile function and slow intra-appendage flow prolong contrast mixing time and cause persistent early-phase low attenuation in the distal LAA; such slow-flow states are well known to promote spontaneous echo contrast and thrombus formation on echocardiography [15,18,19,20]. Liu et al. [21] demonstrated that early-phase LAA filling defects on cardiac CTA were significantly associated with reduced LAA flow velocity and enlarged atrial and appendage volumes. Our observation that a higher O/D ratio (and corrected O/D ratio) was associated with lower LAA emptying velocities measured by TEE supports this hemodynamic interpretation. Given the influence of cardiac output, heart rate, and acquisition timing on contrast opacification, we used the O/D ratio as an objective metric and further corrected it using the LA-HU/DA-HU ratio. In analyses of TEE-derived flow velocity, the corrected O/D ratio showed better consistency with LAA flow velocity, which partly supports the rationale for using the corrected O/D ratio. Moreover, time-resolved CT techniques and estimation of 4D-CT flow have recently demonstrated that CT can quantify transmitral and LAA flow dynamics, further supporting the use of CT not only for anatomical assessment, but also for functional evaluation of LAA hemodynamics [22].

From a clinical perspective, incorporation of CTA-derived LAA metrics with CHA_2_DS_2_-VASc showed a relatively superior C-index, indicating complementary information for thromboembolic risk stratification. While the CHA_2_DS_2_-VASc score remains a robust and widely used clinical tool, it does not incorporate other potential LAA-related risk factors, including morphological features (volume, orifice area, depth, and shape) or dynamic flow disturbances [17,23,24,25,26]. In addition, because the CHA_2_DS_2_-VASc score includes the item of “stroke”, this may introduce temporal bias and inflate the C-index in a cross-sectional analysis, thereby reducing the observable incremental value of additional CTA-derived parameters. Accordingly, our data proposed a hypothesis that corrected O/D may provide complementary discrimination information, particularly in patients with intermediate or borderline risk profiles based on clinical scores alone. Further prospective studies are warranted to verify this hypothesis. A recent study by Nicol et al. [17] demonstrated that, in multivariate analysis, only the presence of pseudo-thrombus within the LAA and the CHA_2_DS_2_-VASc score were independently associated with the risk of stroke, whereas LAA morphology and volume were not independent predictors. These findings suggested that the influence of LAA morphology on the risk of stroke may be indirect, possibly mediated through its impact on intra-appendage flow dynamics.

Nio et al. [13] demonstrated that reduced LAA attenuation on CT correlates with slow-flow states and is frequently detected in patients with acute ischemic stroke, highlighting the potential role of CT as a screening modality in selected stroke populations. Sun et al. [14] showed that two-phase or delayed CT protocols (early plus 1-min and occasional 3-min delayed imaging) substantially reduce false-positive early-phase filling defects by distinguishing contrast stasis from true thrombus, which has direct implications for clinical workflows when CT is used for risk assessment or procedural planning. Compared with prior studies that primarily used qualitative assessments or small cohorts, our quantitative O/D ratio and corrected O/D ratio are reproducible and may be less dependent on single-phase timing. Systematic reviews and registry data suggest that LAA-targeted strategies (occlusion or intensified monitoring) can influence stroke outcomes and that there is substantial practice variability across centers, underscoring the need for objective imaging biomarkers to guide personalized management [27,28].

## 5. Limitations

However, several important limitations and caveats warrant discussion. First, CTA attenuation values are influenced by multiple technical and physiologic factors (contrast injection protocol, cardiac output, timing of acquisition, scanner type, reconstruction parameters), which can introduce measurement variability. We attempted to mitigate some of these confounders by using the O/D ratio and a further correction with LA/DA attenuation, but residual confounding is possible. Second, the retrospective cross-sectional design precludes demonstration of a prospective causal relationship between early-phase LAA opacification and future embolic events; some patients with LAA-PCO may simply have had prior embolic events that altered LAA function. In addition, the composite endpoint combining ischemic stroke and systemic embolism may introduce some heterogeneity. However, because ischemic stroke represented 95.2% of embolic events in our cohort, the composite endpoint primarily reflected stroke events. In our study, imputation of missing data using mean or median values may introduce additional bias, even though variables with substantial missing data (>20%) were excluded. Third, although we showed reproducibility in HU measurements, the lack of a standardized, universally accepted threshold for defining “clinically significant” O/D ratio or corrected O/D ratio limits immediate translation into guideline recommendations. Fourth, analyses involving LAA emptying velocity were based on a small subgroup with limited statistical power and require validation in larger cohorts. Finally, our cohort consisted of patients scheduled for LAA occlusion or ablation at a single tertiary center, which may limit generalizability to broader AF populations.

These limitations suggest clear directions for future research. Prospective, multicenter studies with standardized dual-phase CT protocols (including delayed imaging) are needed to (1) validate optimal quantitative thresholds for O/D ratio and corrected O/D ratio; (2) assess the prospective predictive value of early-phase LAA opacification for incident embolic events; and (3) compare CT-derived functional metrics directly with gold-standard modalities (serial TEE, 4D flow MRI) and clinical outcomes. In addition, advances in time-resolved CT imaging, automated HU-based analysis, and artificial-intelligence-driven quantification may improve robustness and clinical applicability of CT-based LAA functional assessment.

In summary, our study supports the concept that poor distal LAA opacification on early-phase cardiac CTA is an imaging marker of impaired LAA hemodynamics and is independently associated with arterial thromboembolism in AF patients. Quantitative CTA metrics (O/D ratio and corrected O/D ratio) may complement clinical risk scores and help refine individual thromboembolic risk assessment, though prospective validation and standardization are required before routine clinical adoption.

## 6. Conclusions

Poor distal LAA opacification on early-phase cardiac CTA is associated with an increased risk of ischemic stroke and systemic arterial embolism in patients with non-valvular atrial fibrillation. Quantitative CTA-derived metrics—particularly the O/D ratio and corrected O/D ratio—add complementary information to the CHA_2_DS_2_-VASc score for thromboembolic risk stratification. Prospective validation, harmonization of scanning protocols, and establishment of reproducible thresholds are necessary steps before these metrics can be incorporated into routine clinical risk stratification.

## Data Availability

The datasets analyzed in the current study are available from the corresponding authors upon reasonable request.

## Abbreviations

AF, atrial fibrillation; LAA, left atrial appendage; CTA, computed tomography angiography; PCO, poor contrast opacification; AAO, ascending aorta; PA, pulmonary artery; LA, left atrium; DA, descending aorta; HF, heart failure; LV, left ventricle; RV, right ventricle; RA, right atrium; LVEF, left ventricular ejection fraction; hs-TnI, high-sensitivity troponin I; BNP, B-type natriuretic peptide; PLT, platelet; HGB, hemoglobin; WBC, white blood cell; TBIL, total bilirubin; Cr, creatinine; eGFR, estimated glomerular filtration rate; LAA-O, ostium of left atrial appendage; LAA-M, mid portion of left atrial appendage; LAA-D, distal portion of left atrial appendage; HU, Hounsfield unit; O/D ratio, LAA-O HU/LAA-D HU; corrected O/D ratio, (LAA-O/LAA-D)/(LA HU/DA HU).
