# Supplementary material for: Poor Distal Left Atrial Appendage Opacification on Early-Phase Cardiac CTA and Association With Arterial Thromboembolism
Source: Rev Cardiovasc Med. 2026 Jun 25;27(6):47945. doi: 10.31083/RCM47945 (PMC13339168; doi:10.31083/RCM47945)
Supplement: Supplementary file 1 [file 2153-8174-27-6-47945-s1.zip › Supplementary Material.docx]

**Supplementary Table 1.** Univariate logistic regression analysis

| Variable | B | S.E | Wals | Sig | Exp（B） | 95%CI | |
| --- | --- | --- | --- | --- | --- | --- | --- |
|  |  |  |  |  |  | (lower) | (upper) |
| Age | 0.066 | 0.013 | 25.082 | 0.001 | 1.068 | 1.041 | 1.096 |
| Diabetes | 0.526 | 0.266 | 3.901 | 0.048 | 1.693 | 1.004 | 2.853 |
| Hypertension | 0.729 | 0.240 | 9.209 | 0.002 | 2.073 | 1.295 | 3.320 |
| EHRA score | -0.601 | 0.158 | 14.458 | 0.001 | 0.548 | 0.402 | 0.747 |
| CHA₂DS₂-VASc | 0.905 | 0.1 | 81.745 | 0.001 | 2.472 | 2.032 | 3.008 |
| HAS-BLED | 1.770 | 0.194 | 83.517 | 0.001 | 5.872 | 4.017 | 8.584 |
| LA (mm) | 0.05 | 0.018 | 7.769 | 0.005 | 1.051 | 1.015 | 1.089 |
| RA (mm) | 0.035 | 0.015 | 5.849 | 0.016 | 1.036 | 1.007 | 1.066 |
| BNP | -0.001 | 0.001 | 2.785 | 0.095 | 0.999 | 0.998 | 1.000 |
| PLT | 0.003 | 0.002 | 3.658 | 0.056 | 1.003 | 1.000 | 1.007 |
| TBIL | -0.04 | 0.017 | 5.735 | 0.017 | 0.961 | 0.929 | 0.993 |
| LAA-PCO | 0.892 | 0.278 | 10.254 | 0.001 | 2.439 | 1.413 | 4.209 |
| LA HU | 0.004 | 0.001 | 9.364 | 0.002 | 1.004 | 1.001 | 1.006 |
| LAA-O HU | 0.005 | 0.001 | 12.193 | 0.001 | 1.005 | 1.002 | 1.007 |
| LAA-M HU | 0.003 | 0.001 | 6.028 | 0.014 | 1.003 | 1.001 | 1.006 |
| LAA-D HU | -0.002 | 0.001 | 8.364 | 0.004 | 0.998 | 0.996 | 0.999 |
| DA HU | 0.004 | 0.001 | 7.362 | 0.007 | 1.004 | 1.001 | 1.007 |
| O/D ratio | 0.296 | 0.064 | 21.207 | 0.001 | 1.344 | 1.185 | 1.524 |
| Corrected O/D ratio | 0.342 | 0.075 | 20.947 | 0.001 | 1.408 | 1.216 | 1.630 |

LA: left atrium; RA: right atrium; BNP: B-type natriuretic peptide; PLT: platelet; TBIL: total bilirubin; LAA-O: ostium of left atrial appendage; LAA-M: mid portion of left atrial appendage; LAA-D: distal portion of left atrial appendage; DA: descending aorta; HU: Hounsfield unit; O/D ratio: LAA-O HU/ LAA-D HU; Corrected O/D ratio: (LAA-O / LAA-D) / (LA HU / DA HU)

Supplementary Table 2. Correlations between LAA emptying velocity and variables

| Variable | Correlation coefficient | P value | 95% CI lower | 95% CI upper |
| --- | --- | --- | --- | --- |
| CHA_2_DS_2_-VASc score | -0.313 | 0.017 | -0.534 | -0.052 |
| LAA-D HU | 0.418 | 0.001 | 0.171 | 0.615 |
| O/D ratio | -0.291 | 0.027 | -0.517 | -0.028 |
| Corrected O/D ratio | -0.400 | 0.002 | -0.602 | -0.151 |

LAA-D: distal portion of left atrial appendage; HU: Hounsfield unit; O/D ratio: LAA-O HU/ LAA-D HU; Corrected O/D ratio: (LAA-O / LAA-D) / (LA HU / DA HU)

Supplementary Table 3. LAA emptying velocity in different groups categorized by Corrected O/D ratio

| group | Velocity (cm/s) | P value * |
| --- | --- | --- |
| O/D ratio < 1 (n = 21) | 0.519±0.184 | - |
| 1 ≤ O/D ratio < 2 (n = 22) | 0.539±0.178 | 0.719 |
| O/D ratio ≥ 2 (n = 15) | 0.348±0.171 | 0.008 |
| Corrected O/D ratio < 1 (n = 21) | 0.566±0.164 | - |
| Corrected 1 ≤ O/D ratio < 2 (n = 24) | 0.480±0.200 | 0.127 |
| Corrected O/D ratio ≥ 2 (n = 13) | 0.352±0.156 | 0.001 |

*O/D ratio < 1 and Corrected O/D ratio < 1 are reference groups

O/D ratio: LAA-O HU/ LAA-D HU; Corrected O/D ratio: (LAA-O / LAA-D) / (LA HU / DA HU)
